# Nurse educators’ experiences implementing work-integrated learning in the R171 nursing program at a college

**DOI:** 10.4102/hsag.v30i0.2960

**Published:** 2025-12-19

**Authors:** Moloko J. Moremi, Molatelo M. Rasweswe, Tintswalo V. Nesengani, Modiehi H. Legodi

**Affiliations:** 1 Department of Nursing, Faculty of Health Sciences, University of Pretoria, Pretoria, South Africa; 2 Department of Nursing Science, Faculty of Health Sciences, University of Limpopo, Polokwane, South Africa; 3 Department of Human Nutrition and Dietetics, School of Health Care Sciences, Sefako Makgatho Health Science University, Pretoria, South Africa

**Keywords:** experiences, nurse educators, R171 nursing programme, Nursing Program, Nursing College

## Abstract

**Background:**

The global call for nursing education transformation is gaining momentum. South Africa’s R171 nursing programme, revised since 1984, aims to produce generalist nurse practitioners with required competencies. This research aims to explore nurse educators’ experiences in implementing the R171 nursing programme.

**Aim:**

To explore the experiences of nurse educators implementing R171 work-integrated learning (WIL).

**Setting:**

The interviews took place at a nursing college in a private room with no interruptions. Unstructured interviews were used.

**Methods:**

The study utilised a qualitative descriptive phenomenological design to investigate the experiences of nurse educators implementing the R171 nursing programme. The participants were selected through non-probability purposive sampling from the Gauteng College of Nursing (GCON) campuses. The sample size was determined by data saturation. Data were collected through unstructured interviews.

**Results:**

The study identifies five themes: WIL allocation in the R171 nursing programme, challenges faced by nurse educators, consequences of these challenges, identified strengths and recommendations made by these educators for implementing the R171 WIL programme.

**Conclusion:**

The study revealed challenges in student nurse placements, including access to clinical practice areas, discipline time, assessments, staff shortage and resource limitations.

**Contribution:**

The study recommends revising the R171 programme, phasing it out over a year, increasing WIL hours, starting the primary healthcare (PHC) module in the second year, improving infrastructure and re-establishing the Clinical Education and Training Unit (CETU).

## Introduction

In South Africa, a 4-year Diploma in Nursing (R425) was offered since 1984. South African Nursing Council has since revised the nursing curriculum to align with higher education standards 2014:2). The purpose of the revision was to have a nursing programme that produces competent, dedicated generalist nurse practitioners who can demonstrate the required competencies and apply them in diverse healthcare settings.

The R171 nursing programme is a new South African nursing diploma course. This nursing programme is community-based and was implemented for the first time in 2020. The duration of the course is 3 years, including work-integrated learning (WIL). The R171-qualified registered nurse may not accept accountability and duty for overseeing all nursing care at a medical facility, service or establishment SANC 2020.

This new nursing diploma course replaces the 4-year Diploma in Nursing course (R425), which was phased out in 2019 (SANC 2020:5). The R425 nursing programme was a 4-year course leading to general, community, psychiatry and and midwifery nursing. This legacy nursing programme is being phased down in accordance with the Higher Education Qualifications Sub-Framework’s standards, much like all other legacy qualifications in the nation (SANC 2020:1).

The students enrolled in R171 are expected to acquire knowledge and skills in the community to meet the WIL outcomes. The WIL outcomes incorporate rendering community healthcare, palliative care, rehabilitative care and home-based care (SANC 2020:2). The WIL in clinical practice is facilitated by nurse educators in accredited clinical facilities. They also assess the clinical competency for specific skills according to the level and year of study. For WIL to take place, students are placed at the facilities for a certain number of hours that are determined by the SANC (2020:3).

In clinical practice, the students are to correlate theory into practice and gain competency in demonstrating required skills (Mthiyane & Habedi 2018:4). There are challenges facing both the students and educators in implementing WIL for the R171 nursing programme during the beginning phases of course implementation because of limited information prior to the implementation of this programme (Blaauw et al. 2014:2). Hence, there is a need to explore the experiences of the Gauteng College of Nursing (GCON) nurse educators in the implementation of WIL for the R171 programme.

### Background

Globally, there is an urgent call to transform nursing education in order to align the programmes with population health needs. Major shifts in the healthcare system and practice environment in the United States necessitated significant shifts in nursing education. An enhanced educational system is required to ensure that current and future generations of nurses can provide safe, high-quality, patient-centred care in primary, community and public health settings (Gorski, Gerardi, Giddens, Meyer & Peter-Lewis 2015:2).

According to the study conducted in Bahrain by Awadhalla, Al-Mohandis and Al-Darazi (2018:2), there is a need for effective integration between community expectations and clinical education. With the world’s population continuing to rise and illness burdens increasing, there is an urgent need for more qualified healthcare professionals, such as nurses, to improve the quality of nursing and ensure efficient healthcare systems (Bvumbwe & Mtshali 2018:7).

The findings of the study conducted in Bahrain by Awadhalla et al. (2018:2) on clinical education and clinical practice in nursing were ineffectively integrated. Several adjustments were made to improve the quality of clinical learning, including site preparation prior to the start of student teaching and learning, as well as the preparation of registered nurses for preceptorship roles. Students worked directly with patients and the healthcare team. Students are introduced to clinical learning at an earlier stage and are given greater responsibility in a variety of clinical circumstances ranging from simple to sophisticate. The assessment techniques are changed to reflect the intended course objectives and to be more connected with the programme’s competencies.

A study conducted by Bvumbwe and Mtshali (2018:7) mentioned that nursing education in sub-Saharan African nations faces significant problems because of poor infrastructure, a lack of skilled human resources specialists and a lack of material resources. With the world’s population continuing to rise and illness burdens increasing, there is an urgent need for more qualified healthcare professionals, such as nurses, to improve the quality of nursing and ensure efficient healthcare systems. With a focus on community health needs, training initiatives should prioritise competency acquisition through long-term mentorship and supervision, as well as simulation, rather than ad hoc short-term lectures and seminars. Therefore, nursing students must be more prepared to meet the increasing needs of the South African multicultural population while considering the current and future social, cultural and economic factors.

Blaauw et al. (2014:2) further alluded that the restructuring would make curricula more congruent with the knowledge and skills meet the needs of a changing society and economy. However, there are both benefits and challenges that come with the implementation of the new nursing programmes. Benefits include making educational plans more relevant to knowledge and skills to address the issues of an evolving society. Challenges include changing the scope of training and addressing the vulnerability of new qualifications versus the legacy qualifications and the presumption that changing the qualifications will affect the nursing profession (Matlakala 2016:6).

Nursing education and training includes clinical teaching and learning. One of the most significant factors influencing the teaching-learning process in clinical settings is the students’ exposure to clinical learning environments. In nursing education, WIL allows nursing students to correlate theory into practice, as they engage and care for patients, clients, family members and community members in the clinical learning environment. Therefore, nursing students and their clinical lecturers should have access to adequate healthcare services that are accredited by the SANC for nurse training to achieve the programme’s learning objectives (Mthiyane & Habedi 2018:8).

The national strategic plan for nurse education and training (Department of Health [DOH] 2013–2017:22) reveals that there is a shortage of clinical training sites, facilities and environments in many healthcare organisations, which leads to a lack of clinical learning opportunities. There is an absence of student supervision and management. There is also a gap between the skills and competencies of nurse educators and those of nurses in clinical practice. This is exacerbated by the lack of communication between nursing education and practice. The study on the ability of newly qualified nurses to practise independently in different settings found that students are dissatisfied with their clinical facilitation and accompaniment; they lack positive role models, do not apply certain theory in clinical situations, suffer high stress levels and do not feel prepared to fulfil their roles (DoH 2013).

In 2020, the coronavirus disease 2019 (COVID-19) caused a worldwide pandemic. This pandemic led to lockdown restrictions, thereby causing closure to South African schools, including tertiary institutions (*Disaster Management Act 57*, South African Government 2020, COVID-19 regulation, 15 March 2020). The higher education institutions switched to online teaching and learning for both theory and clinical. Many nursing institutions closed their doors to outsiders to help curb the pandemic, meanwhile denying students opportunities to practise (Jamshidi, Molazem, Sharif, Torabizadeh & Najafi, 2016:2).

### Problem statement

The R171 nursing programme was developed and implemented in 2020 to overcome the shortcomings of R425 (SANC 2014:2). However, just like any other new programme, the implementation of the R171 nursing programme has benefits, challenges and uncertainties compared to the legacy curriculum R425 (Poto 2016:5). The uncertainties include outcomes, modules, qualifications, the study content, teaching approach, methods of assessment and clinical competency (Poto 2016:12). According to Matlakala (2016:6), it is because there is a lack of a clear difference between the new and the legacy programmes.

A study conducted by Bvumbwe and Mtshali (2018:7) states that nurse graduates still lack necessary competencies, as a result of poor strategic leadership to drive transformation, unresponsive curricula, nursing faculty shortages and a lack of teaching and learning resources. All of these contribute to inadequate production capacity of training institutions. Effective academic practice partnerships can reduce the theory-practice gap, thereby improving patient safety, reducing medical errors, strengthening practice settings and cushioning faculty shortage.

Matlakala (2016:2) argues that the R171 curriculum needs to be examined and researched further to produce quality, competent healthcare professionals to meet the needs of the country. Hence, the researcher aims to have an in-depth understanding of the clinical nurse educators implementing WIL of the R171 nursing programme in a nursing college.

## Research design and method

A qualitative descriptive phenomenological design was used in this study. A qualitative research strategy is a rigorous, interactive, holistic subjective method of describing life experiences, cultures and social processes from the perspective of the people involved (Gray, Grove & Sutherland 2016:38). A descriptive phenomenological design was used to explore WIL experiences as lived by the nurse educators at GCON when implementing the R171 programme.

The descriptive method was used to describe the experience of clinical nurse educators implementing the R171 WIL programme. The researcher chose this method to discover new knowledge, experience and understanding of human experience from the participants’ viewpoint (Brink et al. 2018:104). The purpose was to explore and describe the experiences of clinical nurse educators implementing the R171 WIL programme in a nursing college.

The explorative method was used to explore the views, feeling and emotions during the interviews to describe the perspectives of participants. The researcher asked participants to relate the experience and also interpret the subjective meaning as well (Fouche et al. 2021:295). The researcher used these methods to collect data relevant to research problem by probing and paraphrasing. Collected data were further explored and interpreted to find the meaning.

In order to understand detailed and insightful information of the phenomenon, a four-step process for descriptive phenomenology was described (Polit & Beck 2017:833). The four steps are bracketing, intuiting, analysing and describing.

The target population for this study was clinical nurse educators working at the selected four GCON campuses who are implementing the R171 WIL programme in 2020. There were representatives from each campus. The recruitment was undertaken through the campus heads, after receiving permission to conduct the study from the National Department of Health, and ethical approval was granted from the University of Pretoria, Faculty of Healthcare Sciences. The participants’ years of experience ranged between 2 and 15 years, as clinical nurse educators were included in this study. The R171 theory nurse educators who were not accompanying students to clinical areas were excluded because the study was focusing on R171 WIL programme:

Purposive sampling was used to select the participants. Data were collected through one-on-one interviews. Prior to the main study, a pilot study was conducted with two clinical nurse educators, each from different nursing campuses, in order to trial the questions and refine the interview skills of the researcher. The two pilot study participants also participated in the main study.

The students’ counselling rooms at each campus were used as venues for face-to-face interviews. For teams’ interviews, the private educator’s offices were used for easy accessibility to the computers. The notice of ‘do not disturb interview in progress’ was displaced at the doors of the rooms to alert people.

Table 1 summarises the details of participants.

**TABLE 1:** Summary of participants’ information.

GCON campuses	Gender	Age (years)	Department of practice	Years of teaching experience	Speciality
**Campus A**					
Participant 1	Female	61	Clinical nurse educator	15	Primary healthcare clinical educator
Participant 2	Female	54	Clinical nurse educator	9	Ethos and professional practice educator
**Campus B**					
Participant 1	Female	45	Clinical nurse educator	3	Primary healthcare (PHC) clinical educator
Participant 2	Female	48	Clinical nurse educator	8	PHC clinical educator
**Campus C**					
Participant 1	Female	35	Clinical nurse educator	4	Nursing unit care clinical educator
Participant 2	Female	58	Clinical nurse educator	15	Primary healthcare clinical educator
**Campus D**					
Participant 1	Female	42	Clinical nurse educator	5	Primary healthcare clinical educator
Participant 2	Female	33	Clinical nurse educator	2 ½	Nursing unit care clinical educator

GCON, Gauteng College of Nursing.

Immediately after a clinical nurse educator indicated their voluntary intention of participating in the study, the researcher held a brief meeting and explained the purpose of the study. The researcher requested each participant to sign an informed consent, and once the researcher received the signed informed consent, an appointment for the interview and venue (either face to face or virtual) was agreed. Verbal consent to audio-record the interview was obtained during the interview. The purpose of recording the interviews was to capture the conversation and transcribe it verbatim prior to data analysis.

The interview time, venue and method were determined by each participant’s preference and availability. Some participants preferred an online interview using technology (Microsoft Teams) or WhatsApp video calls, while some participants preferred a face-to-face interview. In the case of a face-to-face meeting, the required physical distancing of 1.5 m between each other and surface and hand sanitation were adhered to, as per the COVID-19 requirements. The interviews lasted between 30 min and 45 min per participant. The participants were encouraged to speak freely without being coerced. The environments and scheduled time were conducive for the interview to take place without interruptions. The researcher took 6 weeks to complete the interviews, as some nurse educators were busy with their clinical accompaniments at various clinical institutions.

The data analysis followed Colaizzi’s method in order to produce the thematic structure and the exhaustive description of the clinical nurse educators’ lived experiences (Polit & Beck 2017:950). Table 2 summarises Colaizzi’s seven steps of data analysis.

**TABLE 2:** Data analysis steps Colaizzi’s (1978).

No	Steps
1	*Familiarisation:* The researcher familiarised herself with the data by transcribing, reading the transcripts repeatedly while listening and comparing the written data to the audio. The researcher then drew a table manually and labelled it with the research title. The table had different headings that included statements, classification, themes, categories and sub-categories.
2	*Extract significant statements:* On the transcripts, the researcher identified significant statements and words and highlighted the ones with similar meaning using a same-coloured pencil.
3	*Formulating meanings:* From the identified statements, the researcher formulated meanings. A reflective memorandum was used to capture the researchers’ assumptions.
4	*Aggregating formulated meanings into theme clusters:* The researcher identified common meanings and clustered them together into groups to categorise common experiences of participants into themes, categories and sub-categories.
5	*Develop exhaustive descriptions:* Through a synthesis of theme clusters, a description of the lived experience of clinical lecturers implementing R171 programme was formed.
6	*Fundamental structure formation:* The researcher then created the structural essence of the phenomenon from exhaustive descriptions.
7	*Returning to the participants for validation:* The researcher validated the findings by returning them to the participants for feedback.

*Source:* Adapted from Colaizzi, P.F., 1978, ‘Psychological research as the phenomenologist views it’, in R.S. Valle & M. King (eds.), *Existential-phenomenological alternatives for psychology*, pp. 48–71, Oxford University Press, New York.

### Confidentiality

The principle includes an individual’s right to self-determination and full disclosure to determine time, extent and general circumstances under which personal information may be shared or withheld from others (Gray et al. 2017:335). The participants’ right to self-determination was respected. Participation was voluntary, and no one was coerced to participate. The researcher asked the questions relevant to the study and informed the participants of their right to withdraw from the study at any stage without any prejudice.

### Protection of Personal Information Act

This refers to the *Protection of Personal Information* (POPI) *Act (Act No.4 of 2013)*. The *POPI Act* restricts the ill use of personal information by individuals and corporations. This law safeguards all residents of South Africa. Misuse of personal information in a healthcare setting is unethical and unlawful, as it violates patient confidentiality (Buys & Somerall 2018:494). In this study, only information and identifying information essential to the study were captured. Participants signed consent forms for all information captured. Confidential information was limited to those directly involved with the research (Botma et al. 2022:18).

### The principle of beneficence

This principle refers to balancing the risks and benefits of participants (Gray & Grove 2017:208). No physical harm was inflicted to the participants during data collection. The researcher tried to reduce the risks by ensuring that participants were protected from victimisation by maintaining confidentiality of their identity. Some psychological discomfort was experienced because of the types of questions that were asked. The researcher reflected on the feelings and discomfort and reassured the participant of the confidentiality. No one showed emotions that needed referral for psychotherapy.

### Trustworthiness

Quality control for this study followed the Lincoln, Y.S. and Guba, E.G., 2018. Naturalistic Inquiry. Sage Publications.

#### Credibility

According to Polit and Beck (2017:154), credibility establishes whether the research findings represent the voices of the participants and correct interpretation by the researcher. In this study, the researcher prolonged his or her engagement with the participants during the interviews, which lasted 30 min–45 min. Member checking was done, in which the transcripts or raw data were taken back to participants for verifications. An independent coder was used to verify themes and relationships between them. The researcher verified the themes by re-checking the transcripts and re-listened to the audio recordings.

#### Dependability

Dependability refers to provision of evidence such that if the study is repeated in a similar context with similar participants, it will yield similar results (Brink et al. 2018:159). The researcher asked a peer who has passed the nursing master’s degree to follow the process and procedure and determine whether findings are acceptable and dependable. Stepwise replication was used. Independent coding by an experienced coder was used to ensure dependability by categorising the data to establish the framework ideas.

#### Transferability

Transferability is the ability to apply findings to another context. The researcher ensured that the study’s findings informed other potential studies. The research findings can be translated to another context. The researcher documented the database that can be used in another setting (Brink et al. 2018:159). The study findings may be used in other nursing fields and other clinical programme areas.

#### Confirmability

Confirmability is the possibility of data congruency in terms of accuracy, relevance or meaning. It verifies that the findings, conclusions and recommendations are backed up by evidence. The researcher’s inquiries concur. An internal audit can be conducted to confirm the findings (Brink et al. 2018:159).

#### Bracketing

The researcher ignored what she knows about the participants’ experiences, as she is a nurse educator. The researcher kept a reflective journal to ensure authenticity. The researcher also used reflexivity, which is critical thinking used to examine the data (Gray & Grove 2017:545). The researcher used personal experience as a nurse educator to explore personal feelings that may influence the study and integrate understanding into this study.

#### Authenticity

The researcher expressed the feelings and experiences of participants as they occurred (Brink et al. 2018:159). Findings reflected the participants’ voices.

### Ethical considerations

All ethical considerations were adhered to from planning the proposal, implementation, report writing and dissemination of findings (Polit & Beck 2017:727l). Approval to conduct the study was obtained from the Ethics Committee of the University of Pretoria on 11 May 2023 with ethical clearance number 60/2022. In addition, the Gauteng Department of Health granted permission to conduct the study. The researcher requested authorisation from the Gauteng College of Nursing Ethics Committee and permission was granted.

## Results

Table 3 indicates the themes and sub-themes that emerged from the data analysis. Five themes and eighteen sub-themes emerged during data analysis.

**TABLE 3:** Summary of the themes and subthemes.

Themes	Sub-themes
1. Work-integrated learning (WIL) allocation in the R171 nursing programme	1.1 Number of WIL hours
2. Challenges experienced by nurse educators in implementing the R171 nursing programme WIL	2.1 Time period allocated to WIL 2.2 Accessing PHC facilities 2.3 Clinical assessments 2.4 Human resource (shortage of nurse educators and resources) 2.5 Curriculum designs 2.6 Meeting learning outcomes at PHC facilities
3. Consequence of challenges experienced by nurse educators in implementing the R171 WIL nursing programme	3.1 Compromised quality of nursing education 3.2 Consequence for nurse educators and students 3.3 Consequence for the health system 3.4 Compromised learning outcomes
4. Strengths experienced by nurse educators in implementing the R171 WIL nursing programme	4.1 Human strengths 4.2 Institutional strengths
5. Recommendations made by nurse educators in implementing the R171 WIL nursing programme	5.1 WIL placements 5.2 Curriculum review 5.3 Human resource

PHC, primary healthcare.

### Theme 1: Work-integrated learning allocation in the R171 nursing programme

This theme confirms that the clinical nurse educators experienced challenges in allocating the R171 nursing students to the clinical facilities because of limited hours.

#### Subtheme 1.1: Work-integrated learning hours

Challenges were mostly related to a fewer hours allocated to students for the clinical exposure. The R171 programme carries 360 credits. The 360 credits will equal 3600 h over 3 years. Each year, students are required to accumulate 1200 h in both theory and practical (SANC 2013:4). In comparison, the South African hours are perceived to be less when compared to the Australian and USA hours, based on these two studies (Roberts, Kaak & Rolley 2019:6).

Findings from the study conducted in South Africa revealed that for the training requirements for the R683 Bridging Course, student nurses were required to complete a minimum of 2000 h in different WIL specialities compared to 1200 h for the R171 programme (Dormehl 2021:44).

The participants in this study have indicated that the R171 WIL hours are not adequate. This implies that students may not find enough time to practise in order to be competent. Some even compared the R171 with the R425 nursing programme. The R425 programme was regulated to have 50% theory and 50% practical. The R171 programme is a semester programme regulated to do 40% theory and 60% practical (SANC 2014:4):

‘Primary Healthcare equals to Clinical Placement Learning has 43hrs, Clinical Simulation Laboratory has 13hrs, whilst Structured Clinical Guidance has 6.5 per week and Learning for rote training has 40hrs per semester.’ (Campus B, participant 2)

‘I think 144 hours per each module, Primary Healthcare and Nursing Unit Care, which is in the programme guide. This is supposed to cover Clinical Placement Learning, Simulated Direct Learning, Structured Clinical Guidance, and Learning for Rote Training for both.’ (Campus D, participant 2)

The South African Strategic Planning for Nurse Education stipulates that the clinical simulation laboratory learning (CSL) should form 20% of WIL. In this study, most of the participants did not know the exact number of hours allocated to the R171 WIL. It is believed that when students spend a long period in the clinical practice rather than the classroom, there will be an increase in the quality of nursing care and students’ professional satisfaction (Arkan, Ordin & Yılmaz 2018:2).

According to the SANC qualification framework, nursing education and training standards, the changes in the South African nurse training curriculum respond to changing needs, developments, priorities and expectations in health and healthcare (SANC 2013:2), implying that despite the differences between the two programmes, nurses who acquire the knowledge, skills and behaviours from the R171 programme are considered to be well equipped and competent to meet the present and future health challenges, improve health and well-being (DoH 2019:78).

### Theme 2: Challenges experienced by nurse educators in implementing the work-integrated learning R171 nursing programme

The theme highlighted the challenges nurse educators experienced during the implementation of the R171 WIL programme.

**The subthemes identified from this theme are:** 2.1. Time period allocated to WIL. 2.2. Accessing primary healthcare (PHC) facilities, 2.3. Clinical assessments, 2.4. Human resource (shortage of nurse educators), 2.5. Curriculum design and 2.6. Meeting the clinical learning outcomes.

#### Sub-theme 2.1: Time allocated to work-integrated learning

The placement duration in different departments is crucial because it determines the learning outcomes. Students need time to orientate themselves to the environment and the procedures that are conducted in the ward. Poto (2016:83) argues that the nursing students must possess knowledge from every discipline, be involved in problem identification and comprehend how to respond to patient requirements:

‘[*T*]hese students are from matric, they need to be allocated in different wards for clinical learning, a week is not enough, for example, firstly two days they are still on orientation, third and fourth day they are trying to find their foot in the wards and clinics, and then they are moved to another clinic ward.’ (Campus C, participant 1)

Some participants highlighted that if students are placed in one discipline for 2 days, students will not learn anything from that discipline. The participants complained about the shorter accompanying sessions. The time period was also criticised as being too short and inadequate.

#### Sub-theme 2.2: Accessing primary healthcare facilities

The study findings indicated that clinical nurse educators were frustrated because they were given limited institutions in which to place the large number of students. They indicated that some students were repeating the modules, which aggravated the situation. The PHC institutions can only accommodate a limited number of students per period. Most of the community health centres could not take more than three students because of the clinic sizes and lack of infrastructure, thereby compromising student learning. The following quotations confirm their frustrations:

‘But placing students in clinical area, in the clinics in the community health centres, community health Centre are small, we still had R425 students as well, so we struggled to get students into clinics, because they could only accept 2 to 3 students at a time,. Therefore, it was difficult in community health centres.’ (Campus C, participant 2)

‘The experience was a bit frustrating because were given limited space to place our students, remember we had 50 students even more because of those who are repeating some modules. PHC will give us two to three spaces for students.’ (Campus D, participant 2)

The study findings from Poto (2021:100) shared the same sentiments, when it indicated that the lack of transportation to clinical practice, libraries, lack of mentors for students and other issues related to institutional infrastructure and material resources are challenges impacting effective implementation of the new nursing qualifications. Learning takes place when students are placed in a safe welcoming environment.

#### Sub-theme 2.3: Clinical assessments

Clinical assessments are used to evaluate the competency of students. Both formative and summative assessments are conducted to test their clinical competencies. Participants have highlighted the number of challenges in both types of assessments. One of the problems raised is starting the assessments late because of poor planning, inefficient evaluation tools, assessing many students in 1 day and limited time to conduct the assessments. The participants commented as follows:

‘GCON Exams are given to assessors in the morning. Mostly, questions will be wrong and rubrics are incorrect and lecturers have start first by correcting those mistakes, it took us two hours to correct the exams mistakes. I have never opened the exam papers, and they are right, we always have to start correcting the exams first, students waiting until late. If GCON can allow us to open the exams a day before.’ (Campus C, participant 2)

A participant stated that:

‘Due to COVID-19, the PHC assessments were not done in PHC clinics but done in hospitals, this confuses students, as they were expecting to do PHC assessments in clinics and not hospital.’ (Campus A, participant 1)

The study on nurse educators’ experience with the development of student nurses’ clinical reasoning skills concluded that the nursing students are inclined to be confused during the assessments, because the educational goals and expectations are incongruent with each other (Aboshaiqah et al. 2018:13). The learning outcomes do not correlate to the assessment outcomes, which may have affected their performance, their integrity and credibility of assessments. Opening of exams a day before the exam date is compromising the credibility of exams; the quality assurance should ensure credibility of exams is maintained.

#### Sub-theme 2.4: The shortages of nurse educators and resources

Shortage of staff is a worldwide challenge. In reference to nursing education and training, the human resource refers to nurse educators, clinical preceptors and registered nurses who are responsible to guide and supervise students during clinical practice (SANC 2016:1). Nurse educators are expected to accompany students to clinical areas to guide and support them. Poto (2016:42) states that the student is accompanied to the patient or client to observe or practise clinical skills under the supervision of a registered nurse, which could be a nurse educator or a mentor, in order to assist the student to acquire clinical skills (Poto 2016:42). The SANC (2016:3) stipulates that the ratio of nurse educators in a clinical setting (WIL) should be 1:15:

‘We are only 4 nurse educators for 50 students, students performed well with support from lecturers. We drive the whole day around the clinics, which makes it difficult to make 8 hours of SCG. They need to hire more lecturers to give quality time for quality education.’ (Campus B, participant 2)

The study findings revealed that nurse educators are not enough to follow all students because of their large numbers. Students are also scattered over many clinical institutions, sometimes one or two in one institution. There are also missed opportunities for students because of few mentors being available. It is not possible for the nurse educators to be in six clinical areas at the same time.

Preceptors supervise, instruct and support nursing students while they develop the requisite competencies (Aboshaiqah et al. 2018:9). The registered nurses in the clinical setting must be skilled to mentor the students. The study conducted by Hakim (2023:26) indicates that students think that the main cause of clinical issues is a lack of skilled mentors in the clinical setting (Emvula 2016:40). The study further indicates that as a clinician, the registered nurse has a duty to promote student growth and follow them throughout their clinical placement. Student nurses will need the direction and support of registered nurses:

‘The students could not meet their clinical outcome because of shortage of resources. Sometimes we will struggle with the equipment that are limited, so you will find that you don’t even have the machine for blood glucose HGT, and machine is not working in the ward, Dynamaps are not working, they don’t even have sometimes manual baumanometers, they are out of order.’ (Campus C, participant 2)

From the research findings by Emvula (2016:50), the study found that hospitals lack the essential resources for the nursing students to practise. The quantity and quality of service delivery depend on the resources that are available and how well they are allocated.

#### Sub-theme 2.5: Curriculum design

In South Africa, curriculum design is regulated by the SANC (2014:1). The curriculum designers should meet the stipulated guidelines by the SANC and criteria set by the Council for Higher Education (CHE). The nursing education institutions (NEIs) should adhere to a high level of quality assurance. The R171 nursing programme met the requirements to be accredited by the regulating bodies. From the participants’ perspective, they believed that the curriculum needs to be reviewed. This was supported by the following quotations:

‘The course did not take into consideration that we are dealing with people’s life. You can place students for a week if you are doing IT because nobody dies, but with nursing is different, we are dealing with the life of people, and you need to ensure that they are competent.’ (Campus C, participant 1)

‘I think before the programme is approved, we had to check if it will have enough hours, looking at the hours, they are not fitting the course needs. The course needs more hours, if we can add a year to the course. Hours do not tally with the outcomes, we waste time in hours than to look at the psychomotor, and we don’t comply because we said they must have 60% WIL and 40% hour’s theory.’ (Campus C, participant 1)

‘I understand that the course has been aligned with Council for Higher Education [*CHE*] but is not practical and is not working for the students. Because in nursing we are dealing with human life.’ (Campus D, participant 2)

The South African R171 nursing programme mishap in implementation is not unique; a study conducted in Bahrain on the transformation of nursing education revealed that clinical education and learning experiences in nursing, as well as community expectations within the healthcare system, were ineffectively integrated (Awadhalla et al. 2018:2). Several adjustments were made to improve the quality of clinical learning, including site preparation prior to the start of student teaching and learning, as well as the preparation of registered nurses for preceptorship roles.

The participants are convinced that the R171 programme has too much content to cover. Those students do not find time to practise and be competent. The study on transformation of nursing education in South Africa believes that the implementation of this programme is not well coordinated because the introduction of the new curriculum was rushed (Matlakala 2016:5).

The R171 nursing programme guide (2020), approved by SANC and CHE, regulates that the theory should be allocated 40%, while clinical practice should be allocated 60%. This allocation of hours should be implemented properly. There seems to be contradictions, as the theory seems to have more hours than WIL in implementation, as indicated by the participants.

#### Sub-theme 2.6: Meeting the clinical learning outcomes

Students are placed in hospitals in different disciplines such as surgical wards, medical wards, orthopaedic wards, paediatric wards, casualty and so forth, while in PHC they need to meet outcomes in palliative care, home-based care, rehabilitation centres and community healthcare centres.

A study conducted in Saudi Arabia on nurse educators’ experiences with the development of student nurses’ clinical reasoning skills concluded that, to develop nursing competencies, students need high-quality nursing education that gives them the knowledge, abilities and attitudes they need to provide nursing care Aboshaiqah et al. (2018:8).

The study further revealed that clinical learning affects the learning outcomes in the actual world through an interaction network of forces. Students receive clinical training so they can expand their knowledge while working alongside other members of the healthcare team on actual patients (Aboshaiqah et al. 2018:9).

Motsaanaka et al. (2022:9) agree that students draw from their experiences to reach a firm diagnosis of patients’ conditions, determine interventions to reduce medical errors and ultimately improve the patient’s health outcomes. This was supported by the following quotations:

‘Hours are not enough to cover all these procedures. Students are not competent because they do not get enough time to practice. Students struggle to perform procedures because they do not get enough time to practice.’ (Campus A, participant 2)

‘PHC assessment were done in hospitals instead of clinic. Due to Covid 19, the PHC assessments were not done in PHC clinics but done in hospitals. That defeat the purpose of PHC module outcome.’ (Campus D, participant 1)

Nursing practice is more psychomotor practice than knowledge acquisition. Students are expected to master the required skill more than theory. With the limited time, students are inclined to be incompetent and risk patients’ lives. For clinical practice, students are placed at the facilities for a certain number of hours, determined by SANC (2016:1). In the clinical practice, the students are to correlate theory with practice and gain competency in demonstrating required skills (Mthiyane & Habedi 2018:4).

To meet the clinical learning outcomes, students need the right equipment to practise with. Without clinical equipment, there is no WIL. Study findings by Addisie et al. (2022:21) concur with this statement, indicating that for clinical learning to take place, the clinical equipment must be available for proper teaching and learning.

This study’s findings revealed that clinical practice plays a very vital role in the education and training of student nurses; hence, the participants are concerned about the clinical practice of this new programme as compared to the legacy programmes. This study has revealed that in the R171 programme, clinical practice is inadequate. This may compromise the quality of education. Currently, South Africa has a high number of medical lawsuits. Improved quality of nursing care may reduce these lawsuits and improve the quality of life of ordinary citizens. Arkan et al. (2018:6) pointed out that the quality of nursing care and students’ professional satisfaction are both improved when students spend a lot of time in the clinical practice.

The programme curriculum developers did not take into account that the R171 programme is an undergraduate programme that needs enough time to cover all the procedures. A new student from matric needs more time to grasp and practise what is taught before they can be deemed competent.

### Theme 3: The consequences of challenges experienced by nurse educators in implementing the R171 work-integrated learning nursing programme

The themes revealed that poor education and training may result in incompetent nurses, leading to poor quality of care and services.

**The subthemes are:** 3.1. Compromised quality of nursing education, 3.2. Consequence for nurse educators and students, 3.3. Consequence for the health system and 3.4. Compromised learning outcomes.

#### Sub-theme 3.1: Compromised quality of nursing education

Nursing education is expected to provide graduates with knowledge, skills and necessary competencies to provide safe quality care to prepare for their professional practice. Failure to achieve this crucial outcome may bear adverse health consequences:

‘The quality of nursing care, we are not going to get with this type of education. Quality we are not going to get with this course, we are pushing hours more than the competency, we are looking at master plan than the competency.’ (Campus C, participant 1)

‘I do not see quality in the nurses we are producing. I think they really have to review this course; semester modules are not working. No quality of work is done.’ (Campus A, participant 2)

‘In my personal opinion, I do not think we are producing quality nurses. Because you work at limited time, space, you do not go through all their outcomes, we do not get time to emphasise and go through them whilst they are exposed to clinical areas.’ (Campus D, participant 2)

A study conducted in South Africa on nursing education challenges and solutions in sub-Saharan Africa found that nurse graduates still lack necessary competencies as a result of poor strategic leadership to drive transformation, unresponsive curricula, nursing faculty shortages and a lack of teaching and learning resources (Bvumbwe & Mtshali 2018:7).

Jamshidi et al. (2016:11) recommended that one of the crucial elements determining the quality of clinical education is the exposure and preparation of students for the clinical environment. Motsaanaka et al. (2022:8) are in agreement, stating that professional competency is essential because it improves the quality of patient care, which healthcare workers are expected to do ethically and professionally.

Gorski et al. (2015:3) reiterated that to ensure that the present and future generations of nurses can provide safe, high-quality, patient-centred care across all settings, particularly in areas like primary care, community and public health, an enhanced education system is required.

South Africa has a high prevalence of medical health lawsuits. This results from poor medical practice and incompetency among healthcare practitioners. The aim of the United Nations Development Programme (UNDP) *2030 Sustainable Development Goals*, Sustainable Developmental Goal 3 (SDG 3) is to ensure quality health for all (UNDP 2015:1), while the Ten Point Plan goal (DSD 2010) is to improve the quality of care for all South Africans (Vasuthevan & Mthembu 2021:249).

#### Sub-theme 3.2: Consequences of nurse educators and students

Participants verbalised their feeling of frustrations on this course. They feel exhausted and frustrated. Some have burn-out syndrome and also experienced declining job satisfaction. There is a high turnover of nurse educators at GCON, and students are also frustrated. Both students and nurse educators work under pressure. This was supported by the following quotations:

‘The assessment will start late and is not fair for student, they become exhausted and even lecturers. Nobody ever asked us about our input, lecturers are exhausted. The GCON just impose on us.’ (Campus B, participant 2)

‘Lecturers have burn out and leave colleges and go and work somewhere else.’ (Campus C, participant 2)

‘You see PHC procedures are done in the hospital. Assessments are done in the hospital instead of clinics; it removes the PHC story, which defeats the purpose of PHC WIL. Students get confused; you allocate me in the clinic when you have to assess me you take me to the hospital. The assessments are not fair for both students and lecturers. Time is not enough for both students and lecturers.’ (Campus D, participant 1)

‘We are not doing justice to these students. It is as if we are testing if students can work under pressure.’ (Campus C, participant 1)

From the study conducted in Iran on the challenges of nursing students in the clinical learning environment, it was concluded that students cannot learn and develop effectively if the difficulties they encounter in the clinical learning environment are not identified. As a result, their ability to grow and develop will be impacted (Jamshidi et al. 2016:12). Jamshidi et al. (2016:13) further state that the students’ ineffective exposure to the clinical learning environment has raised dropout rates. Because of the difficulties they encounter in the clinical context, some nursing students have given up on nursing as a career.

The findings from this study confirm that nurse educators were frustrated and drained while implementing this programme. They alluded to the fact that they are short-staffed, and they are not given enough support by the GCON management. They are not consulted on the issue of programme implementation including clinical placement. Some felt that the GCON management did not involve them in their decision-making, some felt overwhelmed because of the workload and some felt frustrated because of the way the programme was designed. Some even compared that the R425 was better designed than the R171 programme design.

#### Sub-theme 3.3: Consequence for the health system

The participants complained of overcrowding in the clinical practical areas, especially in the palliative care centres, home-based care centres, rehabilitation centres and clinics. Because of the increasing number of students per intake, this could be worse. There is also going to be a shortage of staff in other disciplines; the R171 nursing programme is aimed at a generalist registered nurse who does not do midwifery and psychiatry. This could cause a severe shortage in those disciplines. This was supported by the following quotations:

‘[*N*]ow we do not have accommodation to place the students to do palliative care, family study and home- based care. We were given comment from the students institutions to place the students for those specific “skills.”’ (Campus D, participant 1)

‘some institutions do not accept students. There are also few palliative care centres which compromised student learning. Some facilities will be overcrowded with “students.”’ (Campus C, participant 2)

The challenges of the R171 nursing programme are going to affect the health system because if the students do not receive quality nursing education and training, time to practise their skills, poor supervision and unable to meet their learning outcomes, they are inclined to be incompetent. This may lead to poor nursing care and many departmental lawsuits.

According to Maphumulo and Bhengu (2019:2), medical, including nursing, negligence lawsuits against the DoH have become more common in South Africa, leading to substantial cash settlements. It is said that if the nurses involved had more realistic exposure to clinical settings, some of these settlements would have been avoided. Registered nurses are required by healthcare systems around the world to possess the necessary knowledge, abilities and attitudes to ensure safe practice (Kapp 2020:20). The study has found that the introduction of the new curriculum was implemented too hastily before proper situational analysis was done.

Bvumbe and Mtshali (2018:35) indicated that the increased student enrolment has led to an overcrowded clinical learning environment and competition among students for learning opportunities. Investment in infrastructure will enable higher-quality education and training to deliver the best education and training. A supportive clinical setting significantly influences the teaching and learning processes of students (Farzi et al. 2018:7).

Motsaanaka et al. (2022:2) agreed that because of the placement of students from various NEIs as well as other various health disciplines, there is an overcrowding of students in the public academic hospitals. The overcrowding results in inadequate clinical learning opportunities for the student nurses, which prevents them from integrating theory into practice, to develop their critical thinking and clinical competency, and this prevents them from advancing their careers (Motsaanaka et al. 2022:3).

This study revealed that there is a shortage of clinical institutions in, which to place the R171 students. Students end up travelling far to access clinical facilities. Their safety is compromised. One participant indicated that the staff in the facilities were confused because they did not know what to do with the students because they were not orientated on this programme (Maphumulo & Bhengu 2019:2).

The National Strategic Plan for Nurse Education, Training and Practice (DoH 2013:36) states that in order to improve clinical education and training, clinical teaching departments must be re-established. Most of the clinical education and training units (CETUs) are not yet established. The question is ‘Are the CETU established, and the preceptors empowered?’

#### Sub-theme 3.4: Compromised learning outcomes

Participants have raised concerns about students not being able to meet their WIL outcomes. This may lead to poor nursing care. This could contribute to student’s drop out and could be detrimental to the healthcare system in South Africa. This was supported by the following quotations:

‘Students are expected to master the skills in short interval, no quality of training. They are concerned with completing their learning outcomes.’ (Campus B, participant 2)

‘Most of the time we are only drilling the students on the procedures they are only going to be assessed on.’ (Campus C, participant 2)

Students are given the learning outcomes that must be achieved when they go to clinical areas. The clinical outcomes are used to assess the competency of students. This study has found that the time allocated to achieve the outcomes is limited. The institutions for WIL practice are few, the number of staff to supervise the students is inadequate and there are limited resources.

Nursing students are said to be having difficulties in completing their needed clinical learning outcomes and required hours to be competent in performing certain clinical skills because nursing education involves cognitive, affective and psychomotor learning domains applied in the healthcare environment (Nashwan et al. 2020:595).

A study conducted on designing competency-based education for underprepared college learners by Gerardi et al. (2016:17) has indicated that even though the students learn the basics of nursing in classrooms and simulation labs, they do not have the time to practise and repeat these skills enough to be fully prepared to enter the clinical practice (Gerardi & Crew 2016:17). Motsaanaka et al. (2022:7) state that student nurses need sufficient clinical learning opportunities to develop and advance their clinical expertise to become independent in professional practice.

This study revealed that the students do not practise the procedures as they are supposed to, but only learn what they are going to be assessed on. From the findings of the study conducted on a theory-practice gap, this may have resulted from inadequate supervision (Kaphagawani 2015:230). Poto (2021:37) highlighted that with the new nursing programme transformation, there is a need for infrastructure and technological upgrades for libraries, computer laboratories, skills laboratories, student housing and dining areas, staff offices, teaching and learning equipment, Internet, Wi-Fi and a student information system to support e-learning.

### Theme 4: Strengths experienced by nurse educators in implementing the R171 work-integrated learning nursing programme

The study revealed the strengths identified from the R171 programme. Subthemes: 4.1. Human strengths and 4.2. Institutional strengths.

#### Sub-theme 4.1: Human strengths

The students are exposed earlier in the first level of study to community services and are able to identify the diseases as they start and are able to prevent the diseases. The R171 is a community-based programme because of the changing needs of the population. In response to the shortage of other members of the health team at the PHC level, nurses’ training, which was previously mostly hospital based, became community based. This allows nurses to meet the needs of patients. The R171 students do not form part of workforce, thus allowing them the opportunity to study (DoH 2013):

‘The type of students we have are at par with university level, they can compete with other students in the universities because of their higher Aps score’. (Campus C, participant 1)

‘Students get to meet the community at earlier stage, unlike in hospital where they are sick, at clinics, you have an opportunity to see patients at early stage. Students are exposed to primary healthcare clinics at first year, this gives them the opportunity to see patient at the acute stage of the diseases and observe the clinical manifestation.’ (Campus C, participant 2)

Poto (2021:36) argued that the nursing programmes were less effective as a result of the dual role of a student serving as an employee who is paid by the DoH. This study revealed that the nurse educators are the drivers of this programme. They own it; they were given opportunities to design the curriculum and to develop it, and now they are also implementing it. They were given a chance to do situational analysis. This study will assist them to evaluate and improve the nursing education and training. Most of them were involved in implementing the previous R425 programme, so they can compare the two programmes, and this will assist them to improve the quality of education and training.

Matlakala (2016:6) further indicates that because this programme is new, there is room for improvement. Already students are working under pressure, and maybe the current situation will enable students to be critical thinkers in order to cope with the challenging situations. This transformation may create chances for academic advancement and life-long learning. The nurse educators are now expected to study further and meet the requirements to teach in higher education institutions, like possessing a master’s degree if you are teaching at a degree level.

#### Sub-theme 4.2: Institutional strengths

The study revealed strengths gained by the institutions while implementing the R171 programme. This was motivated by the participant’s feelings that NEIs can function without being aligned to universities. This was illustrated by the following quotations:

‘the clinical institutions absorbed the preceptors that were hired by the college previously.’ (Campus C, participant 2)

‘We are also guided by the SANC and CHE. The nursing college can now function independently without to be aligned to the universities. They can now make their own decision and can moderate each other. They can also offer bachelor’s degree, which previously they could not.’ (Campus D, participant 1)

Matlakala (2016:6) believed that the restructuring of the new programme would align the curriculum with the knowledge and skills needed to meet the needs of a changing society and economy. Changing the qualification may assist to progress the profession, thereby enhancing the quality of nursing care.

One of the objectives of the National Strategic Plan for Nurse Education, Training and Practice 2012/13–2016/17 (DoH 2013) was to re-establish clinical teaching departments (CETU) at all NEIs or hospitals, supported by clinical preceptors and funding to support the initiative, in order to support clinical education and training. Revitalisation of the CETU and reintroductions of preceptors will assist in supporting and supervision of students. This will help students to meet their learning outcomes and achieve quality of care.

The nurse educators were given the chance to conduct situational analysis on their own. This gave them the opportunities to assess the clinical placements in order to render efficient quality education and training and to comply with legal-ethical aspects of the country.

This initiative of situational analysis also assisted the facilities to comply with both SANC and CHE requirements and align it with the DoH (*National Health Act 63 of 2003*, South African Government 2003).

### Theme 5: Recommendations made by nurse educators in implementing the R171 work-integrated learning nursing programme

Subthemes: 5.1. WIL placements, 5.2. Curriculum review and 5.3. Human resource.

#### Sub-theme 5.1: Work-integrated learning placement

The study highlighted the need for review of the WIL programme. The study pointed out that the programme has less hours compared to the previous R425 programme. Participants suggested that more hours be added to the programme to meet the requirements of SANC of 40% theory and 60% hours of clinical practice (SANC 2014:8). Participants recommended the need for more clinical mentors to supervise and support the students. This was illustrated by the following quotations:

‘There is a need for more hours to be allocated to WIL.’ (Campus C, participant 1)

The study results revealed that the programme has less hours for students to practise. Most of these participants were exposed to R425 nursing programme, so they are inclined to compare the two programmes. A participant indicated that:

‘[*I*]f the students can have mentors in the clinical area in all areas whether in the community clinical area or hospitals, somebody to hold their hand and show them how things are done, I think will succumb vent the issue that the training was not sufficient.’ (Campus C, participant 2)

The SANC (2020:12) recommends that at least 60% of the course’s total time must be spent on practice exercises. The end of course practical must last at least 8 weeks without interruption to allow for the transition to the workplace. Perhaps the SANC, as a regulatory body for nursing education and training, can recommend that the one extra year be added to the R171 nursing programme for WIL after obtaining their qualification and to master their outcomes.

The study recommends that the WIL nursing programme should be given more hours for practice, especially because it deals with human life, to meet the SANC requirement of 40% theory and 60% of clinical practice (SANC 2014:8). This should be practical, not only applied in theory but also have measurable outcomes. More practice will ensure competency. Nursing education is the cornerstone of the nursing profession, and it must strive for excellence as it deals with human life. It is recommended that the WIL competency hours be investigated.

#### Sub-theme 5.2: Curriculum review

Participants in this study expressed their concern on the need to review the curriculum. This was exhibited by their expressions that some modules can be separated to allow students to master the content. This was supported by the following quotations:

‘I think they really have to review this course; semester modules are not working. The course needs to be reviewed, students even leave the course because of workloads. Let the authority resolve these challenges. They need to relook in this course.’ (Campus A, participant 2)

‘If they add, 1 year of internship is not going to solve problems. If first year we do human anatomy and nursing care and then 2nd year we do the primary healthcare maybe we can improve the quality of care.’ (Campus A, participant 1)

‘“The PHC WIL be postponed to 2nd year as there is not enough time for both NUC and PHC WIL.” Both the NUC and PHC are core modules with practical component; they can be separated to allow students to master the clinical outcomes for NUC. NUC is hospital based and PHC is done in clinics.’ (Campus C, participant 1)

Naude and Bezuidenhout (2015:12) maintain that careful monitoring and evaluation of the new nursing qualifications framework will improve the implementation of the new nursing programme. The nurse educators, as the main stakeholders of the nursing programme, should monitor and evaluate the curriculum in order to identify the gaps to improve the quality of education and training.

A study conducted in Lebanon recommended that nurse educators are required to periodically evaluate and analyse education curricula, teaching-learning methodologies and programmes created for new nurses in order to retain professionally qualified nursing graduates and care, as well as promote patient safety (Poto 2021:48).

The study findings support the review of the curriculum by clarifying accreditation requirements and boosting nurse educators’ capacity while retaining quality care, which is key to the success of nursing education’s transformation (Gorski et al. 2015:53).

This study has revealed that because the R171 programme is a new programme, it needs to be reviewed, noting all the challenges that have been raised and try to improve the programme. Every programme needs continuous evaluation to check whether it serves its purpose or not. If it does not meet the goals, there is always room for improvement, and even with this programme, it was clear from the participants’ perspective that it needs to be reviewed.

#### Sub-theme 5.3: Human resources

Participants in this study recommended aspects over staff recruitment, salaries and retention. This was exhibited by their expressions that many nursing staff in clinical areas, as well as lecturers in the nursing training colleges, leave their jobs because of burn out. This was illustrated by the following quotations:

‘I think they should look at the salary’s scales, how they recruit staff members from the clinical areas. Also try to retain staff members as well, lecturers have burn out and leave colleges and go and work somewhere else.’ (Campus C, participant 2)

In South Africa’s public healthcare system, there is a developing shortfall of more than 30 000 professional nurses. Soon, the delivery of public health services will suffer greatly because of the developing nurse shortage. In this regard, 50% of nurses registered with the SANC are approaching retirement age, which is a severe concern for our nation (Basson 2022:1).

There are not enough clinical nurse educator positions in public hospitals to supervise and instruct the large number of nursing students. On the other hand, nurse educators are required to have a master’s degree in order to teach nursing students (Basson 2022:1).

Remuneration is the major factor leading to the shortage of nurses. Many qualified nurse educators apply for the lecturers’ positions; they show interest in education and training but decline the offer because of salary scales. The occupational specific dispensation (OSD) did not cover education and training, so nurse educators are paid less when compared to the other nurses’ specialities.

The Strategic Plan for Nurse Education, Training and Practice (2012/13–2016/17, DoH 2013) states that the OSD must be reviewed to accommodate nurse educators. The objective is to improve the salaries of nurse educators and improve the retention of nurses. With the increasing number of students each year, there is a need to increase the number of nurse educators. If the salary scales are improved and there is a staff development, this may attract new nurses who have just completed their studies to join the nursing education and training and replace the retired nurses.

## Discussion

Recommendations are made in accordance with the themes that emerged during data analysis. The recommendations are as follows:

### Number of work-integrated learning hours

The number of WIL hours seems limited as compared to other countries. The SANC, as the regulatory body, must ensure that the R171 implementers adhere to 60% of WIL as stipulated. The R171 programme implementers should recalculate and implement the required hours to ensure that they are aligned to regulatory authority, that is, the SANC and the CHE. This will ensure that the students achieve their learning outcomes.

### Accessing primary healthcare facilities

The study has found that students have challenges accessing the PHC facilities. Students do not learn much when allocated for few days in clinical practice. Students should be placed in a particular service discipline at least a minimum of 2 weeks before they are moved to another one. Clinical hours should be reviewed and recalculated correctly for implementation. Students should be given enough time in a service discipline to achieve their learning outcomes and master the expected skills. The NEIs should ensure that students have access to PHC facilities by establishing a working relationship with palliative care centres, as most are private institutions.

### Assessments

The study has revealed challenges with assessments, large number of students, time period and assessments validity. Assessments should be given more days. There should be independent examiner from universities to ensure the validity of assessments.

### Time period allocated per facility

Students had challenges with time allocated per facility. The time allocated per facility is short for the palliative care, home-based care and rehabilitation centres. Students do not have enough time for clinical practice. They tend to lack confidence to perform skills, which may result in poor education and training. Students should be given enough time in the facility to practise; this is recommended to be at least 2 weeks. Participants revealed that this course is congested. Nurse educators, curriculum developers and the preceptors must come together to review and correct the mishap of R171 programme to produce quality and competent students.

### Human resource (shortages of nurse educators)

The study has revealed that there is a gross shortage of nurse educators. The nurse educator ratio to students should be 1:15. Presently, the ratio is 1:45–60. The DoH should employ more nurse educators. The OSD of nurse educators should be reviewed to attract applicants.

### Curriculum designs

According to the participants, the curriculum is well designed, but the problem is implementation; the curriculum should be reviewed to ensure that the R171 WIL is implemented correctly. Some participants suggested that the PHC module be postponed to the second level of the R171 programme to allow students time to practise nursing procedures.

### Placements in different clinical facilities

The study has revealed that students are placed for a short period in a discipline, and as such they are unable to grasp the taught skills. Students should be placed for at least a week or two in different wards, like surgical, medical or paediatric wards.

### Meeting learning outcomes at primary healthcare facilities

The study found that students have challenges achieving their learning outcomes. The students have problems accessing the PHC facilities; they stay for short times in the disciplines, and there is a very large number of students. Primary healthcare assessments are done in the hospitals instead of clinics. The curriculum should be reviewed.

### Compromised quality of nursing education

The study identified challenges affecting the smooth implementation of the programme. The identified challenges result in compromised quality of education and training. The curriculum should be reviewed.

## Implications

The researcher believes that the study will benefit nursing education and training and research, the DoH, nurse educators, the nursing profession and the nursing students.

### Benefits to nursing students

The aim of the study is to identify the challenges of nurse educators implementing the R171 nursing programme. The identified challenges will be corrected, which will improve the nursing education and training, resulting in competent nurse educators.

### Benefits to nurse educators

The study has identified the challenges of shortage of nurse educators. The nurse educators are presently overworked. The nursing education directorate should hire more nurse educators to relieve the shortage. The DoH needs to review the salary of nurse educators to attract more nurse educators.

### Contribution to nursing education, training and research

The study has revealed that the curriculum should be reviewed. The identified gaps and challenges that are hindering the smooth implementation of the R171 programme will be resolved. The challenges of assessments, students’ placements, accessibility and shortage of resources will be attended to. The identified challenges are rectified, which may assist in producing quality and competent nurse educators. The identified gaps should be researched to improve the quality of education and training.

### Contribution to healthcare system and the Department of Health

The quality, competent nurses will render quality nursing care, thus reducing the number of lawsuits. The health system will be able to allocate and channel the funds to hire more nurse educators and required resources. The community will benefit as they will get better care from competent nurses. The lives of the community will be improved.

### Contribution to the profession

The curriculum will be reviewed to comply with the SANC and the CHE. This will benefit the profession, as the nursing profession must be monitored and evaluated to ensure they comply. The policy on education and training will be reviewed to comply with professional regulatory bodies. This will ensure that the nursing college is adhering to ethical and professional regulations.

## Limitations

The study was undertaken during the early implementation of the R171 nursing programme. The challenges were identified at an earlier stage to improve the nursing education and training. The study would have been conducted after the first group of students had completed, so that the findings could be used to revise the curriculum.

The other limitation is that the study was undertaken during the COVID-19 restrictions. Most of the interviews were conducted virtually to adhere to the regulations. Therefore, the researcher experienced difficulties to observe some of the nonverbal clues during the interviews online than if they were conducted face-to-face.

The researcher also struggled to access the clinical nurse educators during working hours because of shortage. They were always busy; hence, some were interviewed during weekends and after hours.

## Conclusion

The study explored and described the experiences of nurse educators implementing the R171 WIL programme. The aim was to identify the challenges inhibiting the smooth implementation of the R171 WIL programme to improve the quality of nursing education and training. Data were collected through the media and analysed using themes and subthemes.

The study discovered challenges with placements of student nurses, accessing the clinical practice areas, the time period in the disciplines, assessments, shortage of staff and shortage of resources.

The study highlighted the recommendations that can contribute to and benefit the nursing practice, the profession, the nurse educators, nursing education and training, the DoH, nursing students, the community and nursing research.
